# Optimized feline vitrectomy technique for therapeutic stem cell delivery to the inner retina

**DOI:** 10.1111/vop.12160

**Published:** 2014-03-24

**Authors:** Hari Jayaram, Silke Becker, Karen Eastlake, Megan F Jones, David G Charteris, G Astrid Limb

**Affiliations:** * Ocular Biology & Therapeutics, UCL Institute of OphthalmologyLondon, UK; † Vitreoretinal Service, Moorfields Eye HospitalLondon, UK

**Keywords:** Feline vitrectomy, inner retina, stem cells

## Abstract

**Objective:**

To describe an optimized surgical technique for feline vitrectomy which reduces bleeding and aids posterior gel clearance in order to facilitate stem cell delivery to the inner retina using cellular scaffolds.

**Procedures:**

Three-port pars plana vitrectomies were performed in six-specific pathogen-free domestic cats using an optimized surgical technique to improve access and minimize severe intraoperative bleeding.

**Results:**

The surgical procedure was successfully completed in all six animals. Lens sparing vitrectomy resulted in peripheral lens touch in one of three animals but without cataract formation. Transient bleeding from sclerotomies, which was readily controlled, was seen in two of the six animals. No cases of vitreous hemorrhage, severe postoperative inflammation, retinal detachment, or endophthalmitis were observed during postoperative follow-up.

**Conclusions:**

Three-port pars plana vitrectomy can be performed successfully in the cat in a safe and controlled manner when the appropriate precautions are taken to minimize the risk of developing intraoperative hemorrhage. This technique may facilitate the use of feline models of inner retinal degeneration for the development of stem cell transplantation techniques using cellular scaffolds.

## Introduction

Significant advances have recently been made toward the development of stem cell-based therapies that target conditions affecting the inner retina, such as glaucoma. The successful transplantation of retinal ganglion cell precursors in small animal models^1^ necessitates the development of delivery strategies for the translation of these findings toward human therapies.

**Figure 1:**
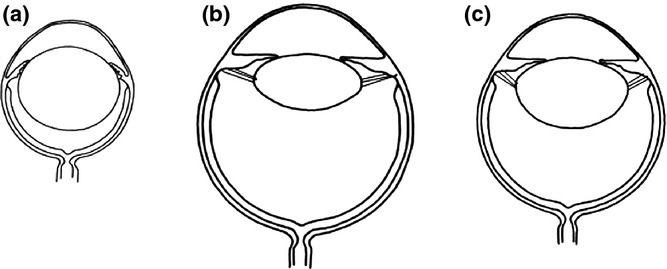
Schematic Diagrams Comparing the Relative Size of Lens and Vitreous Cavity of the Rodent, Human and Feline Eye. (a) The small rodent eye (mean axial length 6.9 mm^16^) has a large crystalline lens and small vitreous cavity. Cells delivered by intravitreal injection will be therefore closely apposed to the inner retinal surface. (b) In comparison, the human lens is relatively smaller compared to the size of the eye as a whole (mean axial length 23.4 mm), with a much larger vitreous cavity.^17^ (c) The feline eye (mean axial length 20.9 mm^18^) although smaller than the human eye has a relatively large crystalline lens, which makes surgical access to the vitreous cavity more challenging. (Illustrations are schematic and not to scale).

A major hurdle yet to be overcome is the ability to achieve a diffuse distribution of transplanted cells over a large area of inner retina in the larger mammalian eye. Intravitreal cell injections into the small rodent eye achieve close apposition of cells to the inner retinal surface due to the large crystalline lens and small volume of vitreous (Fig.1a). However, a similar approach in the larger mammalian eye is likely to prove unsuccessful due to the relatively smaller crystalline lens and larger volume of the vitreous cavity (Fig.1b).

Advances in tissue engineering may help to address this issue, with current work evaluating the potential application of cellular scaffolds to deliver retinal progenitor cells to the subretinal space.^2^ However, a complete posterior vitrectomy would be necessary in order to facilitate the delivery of the cellular scaffold to the inner retinal surface, ensuring that transplanted scaffolds are closely apposed to the host retina and preventing residual vitreous gel acting as a barrier to cell migration.

The cat is the most commonly used animal model in visual prosthesis research and has a well-characterized visual system that is amenable to cortical recording techniques.^3^ With respect to inner retinal pathology, further characterization of a colony of Siamese cats with primary congenital glaucoma^4^ may lead to a suitable model for the future translation of novel therapies involving cellular scaffolds for this condition. However, anatomical considerations make feline vitrectomy a technically demanding procedure. The feline globe is deep set in the orbit making access difficult, and the relatively large crystalline lens (Fig.1c) makes port placement and surgical access to the posterior segment more challenging. In addition, significant intraoperative hemorrhage from the heavily vascularized plexus and anterior ciliary vessels at the pars plana region is a frequent complication.^5^ The purpose of this report is to describe an optimized surgical technique that aims primarily to reduce bleeding and to aid posterior gel clearance to facilitate stem cell delivery to the inner retina using cellular scaffolds.

## Materials and Methods

### Animals

Six female domestic short-haired cats (Isoquimen, Barcelona, Spain) aged between 12 and 16 months were studied. The use of animals in this study was in accordance with the United Kingdom Home Office regulations for the care and use of laboratory animals, the United Kingdom Animals (Scientific Procedures) Act (1986), and adhered to the ARVO statement for the Use of Animals in Ophthalmic and Vision Research.

### Preoperative care

Animals were commenced on oral immunosuppression using prednisolone (1 mg/kg twice daily for the first week, reducing to 0.5 mg/kg twice daily thereafter) and cyclosporine (10 mg/kg twice daily) 2 days prior to surgery with therapy maintained for the duration of the study. Topical atropine sulfate 0.5% eye drops (Minims; Bausch & Lomb, Kingston-upon-Thames, UK) were administered on the evening before and 1 h prior to surgery in order to ensure maximal pupil dilation.

### Transplantation procedure

Anesthesia was induced by intramuscular injection of medetomidine 80 μg/kg (Domitor; Pfizer Animal Health, London, UK), ketamine (5 mg/kg, Narketan; Vetoquinol UK, Buckingham, UK) and butorphanol (0.4 mg/kg, Dolorex; Merck Animal Health, Milton Keynes, UK). This regime provided adequate anesthesia for approximately 60 min. The animals were placed in dorsal recumbency with the head positioned in a semi-circular support to ensure that the cornea was parallel to the operating table, in order to provide an optimal view for subsequent intraocular procedures. Topical anesthesia was provided by application of tetracaine 1% (Minims; Bausch & Lomb) with disinfection of the ocular surface and conjunctival sac achieved with 5% povidone iodine (Moorfields Pharmaceuticals, London, UK). Supplemental oxygen (2 L/min) was delivered under the surgical drape during the procedure.

**Figure 2:**
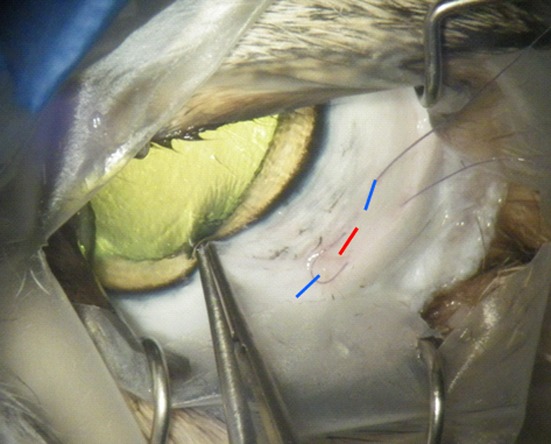
Placement of three temporal sclerotomies. Following cautery to the episcleral venous plexus, a preplaced vicryl suture was placed at the level of the lateral canthal margin 6 mm posterior to the limbus. A central sclerotomy was placed at the center of the preplaced suture (red line) with an infusion line then secured in place. Two additional sclerotomies were placed on either side of the infusion line (blue lines) in exposed sclera at the center of the surgical field.

A limited lateral canthotomy was performed after briefly crushing the lateral canthus with straight artery forceps to achieve hemostasis. Topical epinephrine 0.1% (Moorfields Pharmaceuticals) was applied to the temporal conjunctiva to constrict the episcleral vasculature before performing a three-clock-hour limited peritomy along the limbus with radial cuts to expose the sclera beneath. A pediatric Barraquer lid speculum (Altomed, Boldon, UK) was then inserted to retract the lids, while also reflecting both the conjunctival peritomy and the nictitating membrane to maintain a clear surgical field. Judicious cautery was applied to the episcleral venous plexus before preplacement of a 7/0 vicryl suture through partial thickness sclera at the center of the surgical field 6 mm posterior to the limbus (Fig.2). A 2.5 mm bevelled tip 20-gauge infusion cannula was primed following connection to 500 mL of Ringer's lactate solution containing 0.5 mL of 1:1000 epinephrine to further promote intraoperative hemostasis. A sclerotomy was made with a 20-gauge microvitreoretinal (MVR) blade at the preplaced suture site through which the infusion cannula was inserted and secured. Two further MVR incisions were made on either side of the infusion line in a plane parallel to the limbus in the area of exposed sclera (Fig.2).

Subsequent procedures were performed using a Storz Premiere Microvitrectomy and Phacoemulsification system (Storz; now part of Bausch & Lomb, Rochester, NY, USA). Three of the six animals underwent lensectomy prior to vitrectomy. This was performed using a phacoemulsification probe (30% power, 250 mmHg aspiration) with irrigation fluid directed adjacent to the probe tip using an angulated 20-gauge needle via the two outer sclerotomies. The residual posterior capsule remnants were subsequently removed with the vitrector.

**Figure 3:**
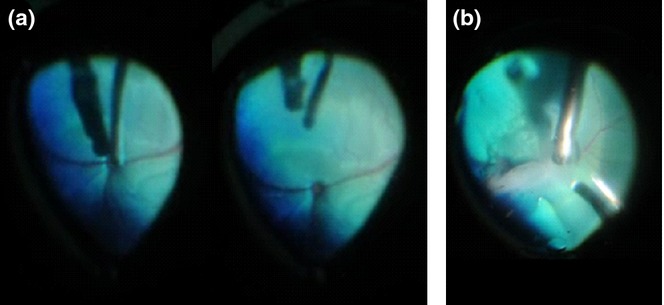
Vitrectomy and Delivery of the Collagen Scaffold Under Direct Visualization. (a) Following core vitrectomy, vacuum was used to induce a posterior vitreous detachment at the optic disk. This was followed by a posterior vitrectomy aided by the use of triamcinolone to visualize residual vitreous cortex in order to aid posterior gel clearance. (b) A collagen scaffold laden with retinal progenitor cells was delivered under direct vision via an enlarged sclerotomy to the inner retinal surface using an 18-gauge cannula.

Where lensectomy was performed, the subsequent vitrectomy could be performed without additional optical correction in the aphakic eye; however, in the lens sparing cases, an external wide angle viewing system with image inverter (BIOM, Oculus, Wetzlar, Germany) was used to visualize the posterior segment. The two ports adjacent to the infusion cannula were used to insert the endoillumination probe and vitrector (Bausch & Lomb). A posterior vitreous detachment was induced (Fig.3a) followed by core vitrectomy (Cut rate 750/min; Aspiration 300 mmHg). Triamcinolone acetonide (Kenalog; Bristol-Myers Squibb, Uxbridge, UK) was used to visualize the residual vitreous cortex in order to ensure that a complete posterior vitrectomy was performed. Fluid–air exchange was performed using a pressurized air line (30 mmHg) connected to the infusion cannula, and a backflush handle attached to a 20-gauge vitreoretinal cannula. Following application of 0.2 U/10 μL chondroitinase ABC (Seikagaku Corporation, Tokyo, Japan) to the inner retinal surface in order to facilitate cell migration and integration,^6^ an 18-gauge cannula was inserted through a sclerotomy to deliver a collagen scaffold with retinal progenitor cells^7^ under direct vision to the inner retinal surface (Fig.3b). The two peripheral sclerotomies were closed using 7/0 vicryl sutures and the infusion cannula carefully withdrawn as an assistant tightened the preplaced suture to ensure a good air fill and prevent hypotony. Finally, the conjunctiva was closed with 7/0 vicryl, the canthotomy closed using 5/0 vicryl, and a subconjunctival injection of dexamethasone and gentamicin administered.

At the end of the procedure, reversal of anesthesia was performed using intramuscular administration of atipamezole 200 μg/kg (Antisedan; Pfizer Animal Health).

### Postoperative care

Animals were given topical atropine sulfate 0.5% daily and dexamethasone 0.1% eye drops (Minims; Bausch & Lomb) twice daily to the operated eye for the first postoperative week, in addition to the oral immunosuppression regime that was continued for the duration of the experiment.

## Results

The surgical procedure was completed successfully in all six animals. Peripheral lens touch occurred in one of the three animals in which lens sparing vitrectomy was performed. Minor bleeding from the sclerotomies was seen in two of six animals, although hemostasis was readily achieved by localized cautery and by temporarily raising the height of the irrigation fluid bag.

No cases of vitreous hemorrhage, severe postoperative inflammation, retinal detachment, or endophthalmitis were observed during postoperative follow-up using slit-lamp examination and indirect ophthalmoscopy.

## Discussion

Early studies into oxygenation of the feline retina initially employed single port combined lensectomy/vitrectomy techniques with little description of operative details or mention of complications.^8^,^9^ This approach evolved to a two-port vitrectomy technique with the use of a sutured infusion line but excluded one quarter of the vitrectomized animals from the study due to vitreous hemorrhages.^10^ Difficulty in surgical access to the deep set feline globe was first highlighted by researchers aiming to deliver a silicone prosthesis into the subretinal space.^11^ This procedure required the removal of a portion of zygoma as well as rotation of the globe with a traction suture to access the temporal sclera. Aside from the invasive nature of the surgery, almost sixty percent of cases were complicated by retinal detachment, although vitreous hemorrhage was not described.

Lens sparing vitrectomy techniques were subsequently reported, which employed a two-port approach in order to facilitate delivery of allografts of neonatal retina^5^ and neural precursor cells^12^ to the subretinal space. This method used four fixation sutures in order to stabilize and rotate the globe in addition to a lateral canthotomy to improve access. Nevertheless, evidence of vitreous hemorrhage was apparent in over seventy percent of animals in the early postoperative period. The extent of bleeding from the sclerotomies was so extensive that closure was not possible in over a third of the animals studied, and massive intraocular hemorrhage leading to termination of the procedure was observed in one case.^5^

In order to successfully deliver cellular scaffolds to the inner retina, it is imperative to minimize the risk of intraocular hemorrhage and achieve complete posterior gel clearance. The use of topical epinephrine to induce localized vasoconstriction, judicious cautery to the episcleral venous plexus prior to creating the scleral incisions, and the use of epinephrine in the irrigating fluid in the present study contributed to the significant reduction in bleeding when compared to previous reports. In addition, raising of the irrigation fluid height to induce a temporary rise in intraocular pressure was a useful adjunct to achieve further hemostasis when required. These adjustments allowed us to perform a safe three-port pars plana vitrectomy in a similar manner to that currently used in human clinical practice.

The use of aqueous triamcinolone acetonide suspension has been reported as an aid to visualize the vitreous and assist in the separation of the posterior hyaloid during vitrectomy.^13^ In the cases we describe, white particles of insoluble triamcinolone remained trapped in any residual gel and were clearly visible in contrast to free-floating particles within the irrigation fluid following removal of vitreous. Residual areas of gel can therefore be readily identified and removed with the vitrector, thus ensuring that complete posterior gel clearance is achieved prior to delivery of the cellular scaffold. Intraoperative triamcinolone has also been reported to inhibit the breakdown of the blood–retina barrier leading to reduced postoperative inflammation.^14^ This also contributes to the creation of a more permissive environment for stem cell transplantation to the inner retina, where triamcinolone has also been shown to be effective in controlling the accumulation of microglia induced by the death of retinal ganglion cells.^15^

The use of air or gas tamponade is routinely employed in retinal detachment surgery in human patients in order to maintain apposition of the reattached retina to the retinal pigment epithelium. In these procedures, the tamponade effect both ensures good apposition of the scaffold to the inner retinal surface facilitating subsequent cell migration and may also serve to reduce the risk of iatrogenic retinal detachment due to entry site breaks.

In summary, three-port pars plana feline vitrectomy may be performed in a safe and controlled manner when the appropriate precautions are taken to minimize the risk of intraoperative hemorrhage. This optimized vitrectomy technique may facilitate the use of feline models of inner retinal degeneration for the development of novel therapies involving cellular scaffolds to deliver stem cells to the inner retina.
